# Non-Redundant and Overlapping Oncogenic Readouts of Non-Canonical and Novel Colorectal Cancer KRAS and NRAS Mutants

**DOI:** 10.3390/cells8121557

**Published:** 2019-12-03

**Authors:** Krizelle Mae M. Alcantara, Joshua Reginald P. Malapit, Ryan Timothy D. Yu, Jose Antonio Ma. G. Garrido, John Paul T. Rigor, Arlou Kristina J. Angeles, Eva Maria Cutiongco-de la Paz, Reynaldo L. Garcia

**Affiliations:** 1 Disease Molecular Biology and Epigenetics Laboratory, National Institute of Molecular Biology and Biotechnology, University of the Philippines Diliman, Quezon City 1101, Philippines; kmmalcantara@yahoo.com (K.M.M.A.); malapit.joshua@gmail.com (J.R.P.M.); ryn_yu@yahoo.com (R.T.D.Y.); anton.garrido@yahoo.com (J.A.M.G.G.); rigor_johnpaul@yahoo.com (J.P.T.R.); arlouangeles@gmail.com (A.K.J.A.); 2 Institute of Human Genetics, National Institutes of Health, University of the Philippines Manila, Manila 1000, Philippines; eccutiongcodelapaz@up.edu.ph; 3 Philippine Genome Center, University of the Philippines System, Quezon City 1101, Philippines

**Keywords:** KRAS, NRAS, colorectal cancer, carcinogenesis, epidermal growth factor receptor pathway

## Abstract

RAS oncogene family members are molecular switches of signaling pathways that control cell growth, proliferation, differentiation, and survival. In colorectal cancer, Kirsten-RAS (KRAS) and neuroblastoma-RAS (NRAS) are the commonly mutated isoforms. Activating mutations in RAS result in cellular transformation independent of upregulated epidermal growth factor receptor (EGFR)-initiated signaling. The present study characterized the functional consequences of non-canonical/novel KRAS and NRAS mutants identified in a targeted next-generation sequencing study of colorectal cancer specimens from Filipino patients. In vitro assays in NIH3T3 cells showed that similar to the canonical KRAS G12D mutant, overexpression of KRAS G12S, A59T, and Y137C, but not NRAS G12D and NRAS A11V, confer higher proliferation and migration rates. HCT116 cells transfected with the novel NRAS A11V and the canonical NRAS G12D, but not the KRAS mutants, display enhanced resistance to apoptosis. All four non-canonical/novel KRAS and NRAS mutants induce gross changes in F-actin cytoskeletal organization and cellular morphology of NIH3T3 cells. Only KRAS G12S and KRAS A59T appear to deregulate extracellular signal-regulated kinase (ERK) and its downstream target ETS transcription factor ELK1 (ELK1). Elucidation of differential effector engagement responsible for the variable phenotypic readouts of the mutants is warranted. If validated by mouse studies and clinical correlates, these can have wider implications in choosing treatment options.

## 1. Introduction

The efficacy of monoclonal antibodies targeting the epidermal growth factor receptor (EGFR) pathway for treatment of colorectal cancer (CRC) depends on predictive biomarkers that can identify likely responders or non-responders to therapy [1]. Downstream of EGFR, the RAS oncogene family members, including the KRAS and NRAS proto-oncogene GTPase isoforms, are critical molecular gatekeepers of cell signaling pathways that control cell growth, proliferation, differentiation, and survival [2]. Gain-of-function mutations in KRAS are reported in 35–40% of CRC cases [3,4], while activating NRAS mutations are detected at a much lower frequency ranging from 3–5% [5]. Mutations in either gene result in hyperactive RAS signaling and constitutive activation of growth signals to downstream effectors and are thus predictive of unfavorable prognosis and poor response to anti-EGFR therapy [6].

Despite the apparent homology and similar structural organization between KRAS and NRAS, numerous studies point to isoform-specific functions and differences in tissues and cancer hallmarks affected. KRAS mutations are more common in pancreatic, lung, and colon cancer whereas NRAS is more commonly mutated in melanomas and acute myeloid leukemia [7]. Further, mutations in analogous hotspot codons (codons 12, 13, and 61) display different frequencies as well as distinct structural and functional defects [8,9,10,11]. KRAS and NRAS mutations are generally considered mutually exclusive but there are recent reports of coexisting mutations in a caecal adenocarcinoma and contiguous tubulovillous adenoma [12]. As a preemptive action, and based on limited clinical evidence, the American Society of Clinical Oncology and the European Society of Medical Oncology have issued revised guidelines for the management of metastatic colorectal cancer patients. RAS mutation profiling now includes codons 12, 13, 59, 61, and 117 for both KRAS and NRAS, and additionally codon 146 for KRAS [13,14]. The non-redundancy of KRAS and NRAS functions, and the high level of tumor heterogeneity observed in CRC, however, suggest wide-ranging clinical and therapeutic implications. At present, functional characterization of these analogous mutations and identification of additional and rare mutations are highly relevant in light of emerging mechanisms of chemoresistance and therapeutic failure.

A retrospective targeted next-generation sequencing study of histopathologically verified malignant CRC patient tumor samples from the University of the Philippines Manila National Institutes of Health [15] identified both canonical and novel or reported but uncharacterized somatic mutations in KRAS and NRAS. One of the mutations occurs within the hotspot KRAS codon 12 (NM_004985.3: c.34G>A), which switches the non-polar glycine residue at position 12 with a polar, uncharged serine residue (p.12G>S; G12S). This mutation has previously been identified as an independent prognostic factor of worse overall survival in CRC patients with colorectal liver metastases [16]. Another missense mutation detected in the colorectal tissue samples occurs within the hotspot exon 2 of KRAS (NM_004985.3: c.175G>A), in codon 59 (p.59A>T; A59T). This mutation lies within the guanosine triphosphate (GTP) binding region of KRAS which implies potential impact on wild-type KRAS protein function. Although included in the list of KRAS mutations to account for, its utility as a negative predictor of response to therapy is not firmly established. A case report of a metastatic CRC patient with this mutation shows an apparent response to the anti-EGFR panitumumab, based on limited radiographic and carcinoembryonic antigen biomarker responses [17]. The functional consequences of these two KRAS mutations are yet to be evaluated, although the Catalogue of Somatic Mutations in Cancer database (COSMIC db) gives them a FATHMM [18] prediction score of 0.98, thus classifying them as both deleterious and pathogenic. Another putatively activating missense mutation was also detected within KRAS exon 3 (NM_004985.3: c. 410A>G) in codon 137 (p.137Y>C; Y137C) which was not found to be reported in COSMIC db and is thus deemed novel. Meanwhile, analysis of NRAS sequences revealed a mutation in codon 11 (p.11A>VA11V). This mutation (NM_002524.5: c. 32C>T) has not been previously reported in the literature and is hence considered novel, with its phenotypic consequences and functional impact on NRAS protein function yet to be determined.

This study reports the functional characterization of the non-canonical KRAS G12S and KRAS A59T mutations, and the novel KRAS Y137C and NRAS A11V mutations identified in Filipino colorectal cancer patient tumor samples. Their effects on cell proliferation, apoptosis, migration, cytoskeletal organization, and gross morphology, as well as their ability to promote epithelial-mesenchymal transition (EMT) and activate the mitogen-activated protein kinase (MAPK) response pathway were assessed. Divergent and overlapping oncogenic properties of the KRAS and NRAS variants were observed and can have therapeutic implications in patients with heterogenous tumors harboring mutations in one or both isoforms. Translatability of these into negative predictive markers for response to anti-EGFR therapy, however, awaits validation from clinical studies involving patients with the requisite mutational profile.

## 2. Materials and Methods

### 2.1. Site-Directed Mutagenesis and Generation of KRAS and NRAS Mutant Constructs

The open reading frame (ORF) fragments of wild-type KRAS isoform b (NM_004985.3) and KRAS G12D canonical mutant variants [19], and the wild-type NRAS isoform 1 (NM_002524.5) and NRAS G12D canonical mutant variants (Yu, R.T and Garcia, R., unpublished) used in this study are available in the laboratory and were previously cloned into the pTargeT^TM^ mammalian expression vector. Site-directed mutagenesis was performed with the pTargeT-wild type (WT) KRAS construct as a template to generate the novel KRAS mutants, using primers listed in Table 1. The pTarget-WT NRAS construct was used as a template for site-directed mutagenesis to generate the NRAS variants used in this study. The primers used are listed in Table 2.

The mutant KRAS and NRAS variant fragments were amplified in a polymerase chain reaction (PCR) mixture containing a final concentration of 1X PCR buffer (Titanium^®^ Taq PCR buffer, Clontech Laboratories, Inc., Mountainview, CA, USA), 0.125 μM of each deoxynucleoside triphosphate (Promega, Madison, WI, USA), 2 μM each of the forward and reverse primers, 1U Taq polymerase (Titanium^®^ Taq polymerase, Clontech Laboratories, Inc.), and 50 ng of the pTargeT-WT KRAS or pTargeT-WT NRAS template. Amplification was performed with an initial denaturation temperature of 94 °C for 5 min, followed by 25–30 cycles of denaturation at 94 °C for 30 s, annealing at 55 °C for 30 s, and extension at 72 °C for 30 s, with a final extension step at 72 °C for 10 min. The amplified 5‘- and 3‘-fragments of each KRAS and NRAS mutant were then used as templates (25 ng of each fragment) for overlap-extension PCR to generate the full-length KRAS and NRAS mutant coding sequences, using the wild-type KRAS or NRAS outer F and R primers and the same PCR conditions stated above. The amplified products were cloned directly into the pTargeT^TM^ vector via TA-cloning. Cloning orientation and sequence identity were confirmed by Sanger sequencing.

### 2.2. Cell Culture and Transfection of NIH3T3 and HCT116 Cells

The murine embryonic fibroblast cell line NIH3T3 and the human colorectal carcinoma cell line HCT116 were sourced from the American Type Culture Collection (Manassas, VA, USA; Cat. No. CRL-1658 and CCL-247). NIH3T3 cells were cultured in Dulbecco’s modified Eagle’s medium (DMEM; Gibco; Thermo Fisher Scientific, Inc., Waltham, MA, USA) supplemented with 10% newborn calf serum (NBCS; Gibco; Thermo Fisher Scientific, Inc.), 100 U/mL penicillin/streptomycin, and 3.7 g/L sodium bicarbonate. HCT116 cells were cultured in Roswell Park Memorial Institute 1640 medium (RPMI-1640; Gibco; Thermo Fisher Scientific, Inc.) supplemented with 10% fetal bovine serum (FBS; Gibco; Thermo Fisher Scientific, Inc.), 50 U/mL penicillin/streptomycin, and 2.0 g/L sodium bicarbonate. All cells were maintained in a humidified incubator at 37 °C with 5% CO_2_. NIH3T3 cells have routinely been used for characterizing RAS oncogenes and their mutant variants, given the cell line’s distinct advantage of not requiring complementary cooperative mutations for oncogenic transformation to manifest [20]. For other cancer hallmark assays, especially those requiring an epithelial phenotype, HCT116 was used. Although a cancer cell line with a KRAS G13D mutant background, its oncogenic phenotype can still be modulated with transfected oncogenes [21].

NIH3T3 cells were seeded for assays in 12-well plates at 40,000 cells/well, while HCT116 cells were seeded for assays in 12-well plates at 300,000 cells/well to achieve 80–90% confluence upon transfection. NIH3T3 cells were transfected with 1 μg pTargeT^TM^ constructs using FuGENE^®^ HD transfection reagent (Promega, Madison, WI, USA). HCT116 cells were transfected with 2 μg pTargeT^TM^ constructs using Lipofectamine^®^ 2000 (Invitrogen; Thermo Fisher Scientific, Inc., Carlsbad, CA, USA). Control setups were transfected with an equivalent amount of a mammalian reporter vector expressing the green fluorescent protein ZsGreen1 (pmR-ZsGreen1, Clontech Laboratories, Inc., Mountain View, CA, USA) to assess transfection efficiency by fluorescence microscopy. A transfection efficiency of 70% and 80% was routinely observed for NIH3T3 and HCT116 cells, respectively. For the morphological characterization experiments, the pTargeT^TM^ constructs were co-transfected with pmR-ZsGreen1 at a vector ratio of 1:3 (pmR-ZsGreen:pTargeT) to allow the observation of cells that had high probabilities of being transfected and are overexpressing the KRAS and NRAS variants. The amounts and ratios of plasmid constructs and of FuGENE^®^ HD or Lipofectamine 2000 were optimized to achieve at least 70% transfection efficiency between 24 and 72 h post-transfection for all morphological, functional, and molecular characterization experiments.

### 2.3. Cell Proliferation Assay

NIH3T3 cells were seeded in 96-well plates at 2500 cells/well and were transfected 24 h after seeding with 200 ng of each pTargeT^TM^ construct in triplicates. Transfected cells were maintained in DMEM media supplemented with 2.5% NBCS. At 48 and 72 h post-transfection, the number of metabolically active cells per setup was measured after the addition of 10 µL CellTiter 96^®^ Aqueous One-Cell Proliferation Assay Reagent (Promega) per well, and incubation at 37 °C until color development. Absorbance values of each setup were measured at 460 nm with a colorimetric plate reader (FLUOstar Omega Microplate Reader, BMG LABTECH, Cary, NC, USA). Cell counts were calculated from a standard curve generated using serial dilutions of an untransfected cell suspension and by plotting the number of cells vs. A_460_. Mean cell counts were calculated per setup for each time point.

### 2.4. Caspase 3/7 Assay

HCT116 cells were seeded in 96-well plates at 10,000 cells/well and transfected in triplicates, as described above. Transfected cells were incubated in RPMI media supplemented with 4% FBS alone, or with 6 mM sodium butyrate for induction of apoptosis. Ten microliters of Caspase-Glo^®^ 3/7 Assay reagent (Promega) was added to each well at 24 h post-induction. The plates were incubated at ambient temperature for 2 h with gentle shaking. Mean luminescence readings per setup was measured with the FLUOstar Omega Microplate Reader.

### 2.5. Wound Healing Assay

NIH3T3 cells were seeded in 12-well plates and transfected as described above. A thin artificial wound was created on the transfected cell monolayers at >90% confluence using sterile toothpicks. Cells were afterwards maintained in DMEM media supplemented with 2.5% NBCS. Wound closure was monitored via time-lapse microscopy for a period of 20 h, capturing the same field of view per well per setup at 1 h intervals at 20x magnification using an Olympus IX83 inverted fluorescence microscope (Olympus Corporation, Tokyo, Japan). Percent open wound area was analyzed using the TScratch software [22]. A linear graph of % open wound area vs. time was generated per setup, and the slope of each line equation was obtained to determine the rate of wound closure. The average rate of three field views per setup was plotted and statistically analyzed.

### 2.6. Western Blotting

The rabbit monoclonal anti-E-cadherin (24E10) (1:5000; Cat. No. 3195S), mouse monoclonal anti-N-cadherin (13A9) (1:5000; Cat. No. 13A9), rabbit monoclonal anti-vimentin (DZ1H3) XP^®^ (1:1000; Cat. No. 5741S), rabbit anti-p44/42 MAPK (Erk1/2) (137F5) (1:1000; Cat. No. 4695S), rabbit anti-phospho-p44/42 MAPK (Erk1/2) (Thr202/Tyr204) (1:1000; Cat. No. 9101S), and mouse monoclonal anti-GAPDH (14C10) (1:10,000; Cat. No. 2118S) antibodies were from Cell Signaling Technology (Danvers, MA, USA). The goat anti-mouse IgG (H+L) (1:5000; Cat. No. 31430) and goat anti-rabbit IgG (1:10,000; Cat. No. 31460) secondary antibodies conjugated with horseradish peroxidase were from Invitrogen (Thermo Fisher Scientific, Inc.).

NIH3T3 cells were harvested 48 h post-transfection. Total protein was extracted using radioimmunoprecipitation lysis buffer (150 mM NaCl, 1.0% IGEPAL^®^ CA-630, 0.5% sodium deoxycholate, 0.1% SDS, 50 mM Tris, pH 8.0) (Sigma-Aldrich Corp.; Merck KGaA, St. Louis, MO, USA) supplemented with protease inhibitors (104 mM AEBSF, 80 μM aprotinin, 4 mM Bestatin, 1.4 mM E-64, 2 mM leupeptin, and 1.5 mM pepstatin A) (protease inhibitor cocktail; Sigma-Aldrich Corp.) and quantified through the bicinchoninic acid assay (BCA) method. For polyacrylamide gel electrophoresis, 30 µg of total protein per setup was loaded into Any kD™ Mini-PROTEAN^®^ TGX Stain-Free™ Protein Gels (Bio-Rad Laboratories, Inc., Hercules, CA, USA) and blotted onto polyvinylidene difluoride (PVDF) membranes. The membranes were blocked for 1 h at room temperature with 5% *w*/*v* bovine serum albumin, heat shock fraction (Sigma-Aldrich Corp.) in 1 X Tris-buffered saline (TBST; 20 mM Tris, 150 mM NaCl, 0.1% Tween 20), and then probed overnight at 4 °C with the primary antibodies described above. After washing thrice with 1 X TBST, the membranes were incubated with the appropriate secondary antibodies for 1 h at room temperature. Signals were developed with enhanced chemiluminescence substrate and imaged using the ChemiDoc Touch Imaging System (Bio-Rad Laboratories, Inc.) using optimal exposure settings. Gene expression levels were obtained by densitometric analysis of digitized band intensities normalized against Glyceraldehyde 3-phosphate dehydrogenase (GAPDH) or total protein loaded in stain-free gels, using GelQuant.NET software (v1.8.2. Biochemlabsolutions, University of California, San Francisco, CA, USA) provided by biochemlabsolutions.com. Total protein loaded in stain-free gels has been reported to provide superior accuracy and reliability in protein semi-quantification compared to commonly used housekeeping genes and was thus also used for protein expression normalization in this study to support our data [23,24].

### 2.7. Actin Cytoskeleton Staining

NIH3T3 cells were seeded at 8000 cells/well in Millicell^®^ EZ 8-well chamber slides (Merck KGaA, Darmstadt, Germany) and transfected with 600 ng of each pTargeT^TM^ construct 24 h after seeding. Transfected cells were fixed with 4% paraformaldehyde at 48 h post-transfection for 20 min on ice, then permeabilized with 0.1% Triton X-100 in 1X PBS for 15 min at room temperature. After washing with 1X PBS, cells were blocked with 1% BSA in PBS for 20 min at room temperature, and then incubated in a 1:100 dilution of tetramethylrhodamine-conjugated phalloidin (Invitrogen; Thermo Fisher Scientific, Inc.) in 1X PBS for 1 h at room temperature with gentle shaking. The cells were again washed with 1X PBS before counterstaining the nuclei with Hoechst 33258 (1 µg/µL) for 5 min at room temperature. After the final washing step in 1X PBS, the cells were mounted in SlowFade^TM^ Diamond antifade mountant (Invitrogen; Thermo Fisher Scientific, Inc.) and were visualized under an inverted fluorescence microscope (IX83, Olympus Corporation), using a red fluorescent filter (λex/λem: 490/525 nm) to visualize filamentous actin structures, and a blue fluorescent filter (λex/λem: 355/465 nm) to visualize the nuclei.

### 2.8. Observation of Gross Morphology

NIH3T3 cells were seeded at 10,000 cells/well in 24-well plates and co-transfected with 500 ng of each pTargeT^TM^ construct together with 100 ng of empty pmR-ZsGreen1 vector 24 h after seeding. Morphological appearance (i.e., size, refringency, presence of filopodia, presence of lamellipodia, and depolarization) of transfected fibroblasts were examined under an inverted brightfield microscope (Olympus IX51, Olympus Corporation) 72 h post transfection. To quantitatively compare the transforming effect on cellular morphology by the different variants of KRAS and NRAS, the percentage of cells exhibiting transformed characteristics was determined for each transfection setup. Each transfected well was viewed in three different fields under 40x magnification. Using the Fiji image processing software (v1.52i, University of Wisconsin-Madison, Madison, WI, USA) [25], fibroblasts with aberrant morphology were counted for each documented field. A total cell count per view was also performed. The mean percentage of morphologically transformed cells was then computed for all three fields of view and statistically compared among all setups.

### 2.9. ELK-TAD Luciferase Reporter Assay

To measure the ability of KRAS and NRAS variants to activate the mitogen-activated protein kinase (MAPK) response pathway, ETS domain transcription factor ELK-1-responsive luciferase reporter HEK293 cells (Signosis Inc. Silicon Valley, San Francisco, CA, USA; Cat. No. SL-0040-FP) were seeded at a density of 10,000 cells/well in 96-well plates. This cell line derived from human embryonic kidney stably expresses the firefly luciferase reporter gene under the control of the ELK1 response element, Gal4-upstream activating sequence (UAS), along with a fusion protein linking the transactivation domain (TAD) of ELK1 to Gal4. HEK293 cells were maintained in DMEM supplemented with 10% FBS and 100 U/mL penicillin/streptomycin and kept in a humidified incubator at 37 °C with 5% CO_2_.

HEK293 cells were transfected with 200 ng of each pTargeT^TM^ construct 24 h after seeding. Media was changed to DMEM supplemented with 4% FBS 24 h post-transfection to deprive the cells of serum that can mask measurable effects which can be attributed to the KRAS or NRAS mutations. At 48 h post-transfection, cells were lysed through the addition of 20 µL of Passive Lysis Buffer (Promega Corporation; Cat. No. E1941) per well and incubation for 20 min at room temperature. Cell lysates were transferred into opaque 96-well plates prior to the addition of 100 µL of the Luciferase Assay Reagent (Promega Corporation; Cat. No. E1483). Luminescence signals per well were measured using a plate reader (FLUOstar Omega Microplate Reader, BMG LABTECH) under the pre-set Firefly luciferase protocol settings.

Parallel wells were also transfected with each pTargeT^TM^ construct to obtain the relative cell count through fluorescent staining of DNA for normalization of luciferase expression. Media was removed at 48 h post-transfection and replaced with 100 µL of CyQuant NF reagent from the CyQUANT NF Cell Proliferation Assay kit (Thermo Fisher Scientific Inc.; cat. no. C35007) per well. Plates were then incubated at 37 °C for 1 h prior to measuring fluorescence readings per well using a plate reader (FLUOstar Omega Microplate Reader, BMG LABTECH) with an excitation wavelength of 485 nm, and emission detection at 530 nm. Luminescence readings from the luciferase assay were then normalized against fluorescence readings indicating relative cell count per setup and statistically analyzed.

### 2.10. Bioinformatics-Based Analysis of Potential Functional Impact of KRAS and NRAS Mutations

The predicted effects of each non-canonical/novel RAS mutation were first analyzed using the Polymorphism Phenotyping (POLYPHEN-2; version 2, Harvard Genetics, Cambridge, MA, USA) [26], Sorting Intolerant from Tolerant (SIFT; version 5.2.2, Bioinformatics Institute, Singapore, Republic of Singapore) [27], and Align GVGD (Huntsman Cancer Institute, University of Utah, Salt Lake City, UT, USA) [28] prediction tools.

The impact of each non-canonical and novel mutation on KRAS and NRAS protein structure was assessed by building homology models recapitulating each mutation, using the solved 3D structure of human KRAS Q61H (PDB:3GFT) and wild-type HRAS (PDB:5DB30) as reference for the KRAS variants, and human NRAS (PDB:5UHV) as reference for the NRAS variants. Each KRAS mutant model was then built using the Accelrys Discovery Studio Client 2.5 (Dassault Systèmes BIOVIA, San Diego, CA, USA) Homology Modeler (HM) protocol, and the global main chain root-mean-square distance (RMSD) and Overlay Similarity (OS) of each mutant model was computed based on structural superimposition with KRAS WT. On the other hand, the NRAS mutant models were built using the SWISS-MODEL web-based client (https://swissmodel.expasy.org/). The global main chain RMSD and OS of each mutant model was computed based on structural superimposition with NRAS WT using the BIOVIA Discovery Studio Visualizer (Dassault Systèmes BIOVIA).

Additionally, the effect of the KRAS mutations on GTP binding was assessed using the LigandFit docking module. The LigandFit docking algorithm involves the need to define binding sites of the KRAS protein based on known interacting amino acids. Monte Carlo-based methods to search for optimal ligand conformations were then done upon the initiation of the docking protocol, after which ligand fitting with GTP was carried out. Best-fit docked models of each KRAS mutation were selected based on the model with the highest Dock Score, and the binding energies of each model were compared among mutants.

### 2.11. Statistical Analysis

All data were statistically analyzed using unpaired two-tailed t-test to measure differences between two setups. Analysis of variance with post-hoc Tukey’s honest significant difference (HSD) was used to test significant differences between multiple setups. Data from all quantitative experiments were presented as mean ± standard error. In all tests, significance value was defined as * *P* < 0.05; ** *P* < 0.01; and *** *P* < 0.001.

## 3. Results

### 3.1. Cells Expressing Non-Canonical/Novel KRAS Mutants Exhibit Increased Proliferation Rates

**Figure 1:**
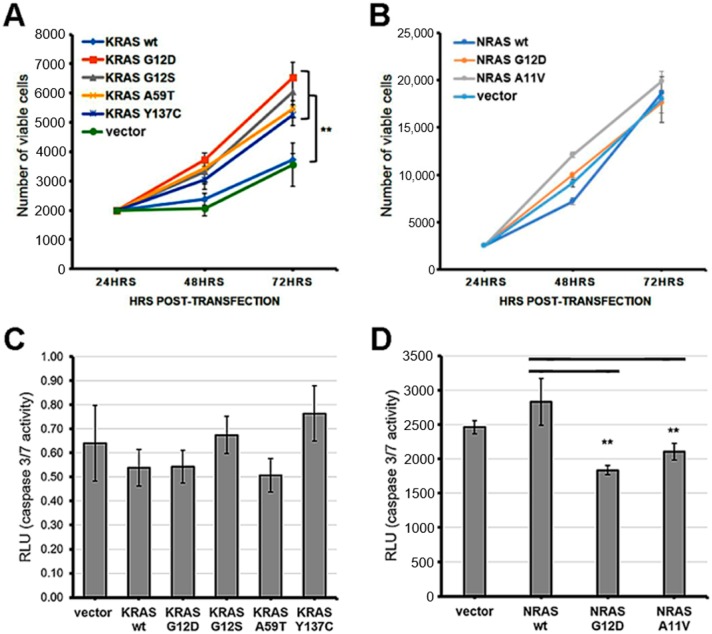
Expression of KRAS mutants enhanced cell proliferation while overexpression of NRAS mutants enhanced apoptosis resistance in NIH3T3 cells. (**A**) Proliferation rate of NIH3T3 cells transfected with empty vector, wild type KRAS or KRAS mutants; (**B**) Proliferation rate of NIH3Te cells transfected with empty vector, wild type NRAS or NRAS mutants; (**C**) Caspase 3/7 activity in NIH3T3 cells transfected with empty vector, wild type KRAS or KRAS mutants; (**D**) Caspase 3/7 activity in NIH3T3 cells transfected with empty vector, wild type NRAS or NRAS mutants. Data presented are representative of three independent trials in triplicates and expressed as mean (standard deviation). ** *P* ≤ 0.01.

To determine whether the non-canonical/novel KRAS and NRAS variants promote cell proliferation, NIH3T3 cells transfected with each mutant were seeded in triplicates and in equal cell numbers onto 96-well plates. Expectedly, KRAS mutants maintained at high serum concentration (10%) showed similar numbers of viable cells upon quantification with a colorimetric reagent at 48 h and 72 h post-transfection (Supplementary Figure S1A), due to full mitogenic stimulation masking possible effects of activating mutations. In contrast, under low serum conditions (2.5%), cells overexpressing the non-canonical/novel KRAS mutants G12S, A59T, and Y137C all displayed a significant increase in cell number at 72 h post-transfection compared to wild-type KRAS and empty vector controls (Figure 1A). Note that as previously documented, overexpression alone of wild type KRAS in NIH3T3 cells does not produce an oncogenic phenotype, although it may in human cells [29,30,31,32,33]. On the other hand, similar to the canonical NRAS G12D mutant, no significant differences in proliferation rates were observed in NIH3T3 cells overexpressing the novel variant NRAS A11V compared to controls in either high serum (10%; Supplementary Figure S1B), or low serum conditions (2.5%; Figure 1B).

### 3.2. The Rare Mutant NRAS A11V Confers Increased Resistance to Apoptosis

The non-canonical/novel KRAS and NRAS mutants were also assessed for their potential to resist apoptosis in colorectal cancer cells. HCT116 cells overexpressing each variant were treated with the apoptosis inducer sodium butyrate (NaB) at 24 h post-transfection under low serum (4%) conditions. Normalization of relative luminescence units was performed against parallel transfected setups maintained in low serum (4%) medium without NaB to account for variations in viable cell numbers. The results showed comparable caspase 3/7 activities among all KRAS mutants compared to wild-type and empty vector controls at 20 h post-treatment with NaB (Figure 1C). In contrast, the novel mutant NRAS A11V exhibited significantly decreased levels of active caspase 3/7 compared to controls, similar to caspase 3/7 activities measured in setups transfected with the canonical variant NRAS G12D (Figure 1D). These results indicate that the novel NRAS A11V variant, but not the KRAS G12S, A59T, and Y137C mutants, inhibits caspase 3/7 activation and promotes resistance to apoptosis in a colorectal cancer cell background.

### 3.3. KRAS Mutants G12S, A59T, and Y137C Promote Cell Migration

**Figure 2:**
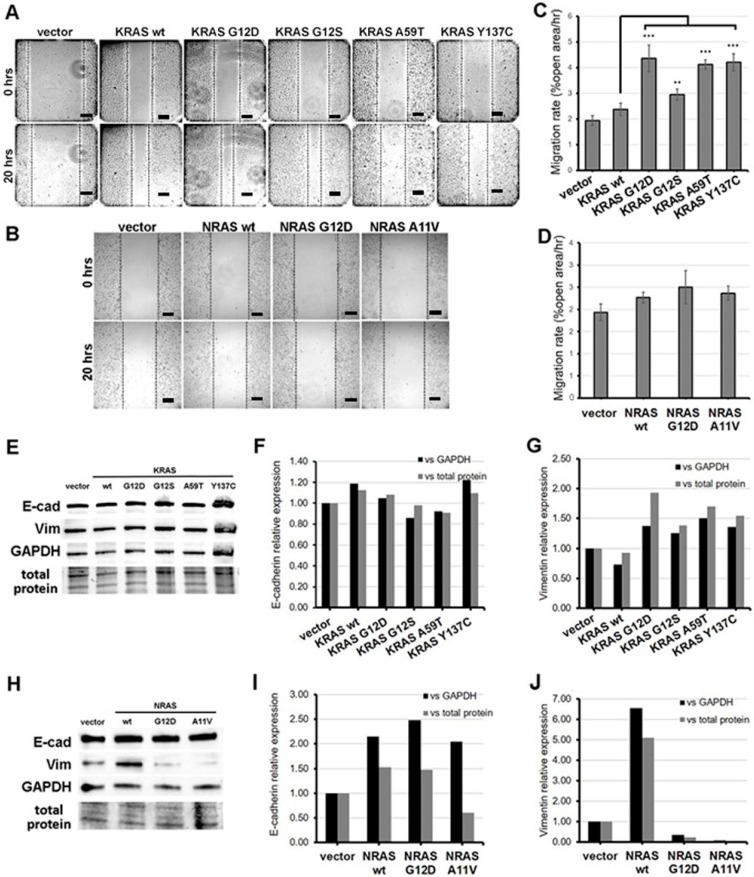
Expression of KRAS mutants enhanced the motility of NIH3T3 cells. (**A**,**B**) Representative micrographs of NIH3T3 cells transfected with (**A**) KRAS and (**B**) NRAS constructs after scratching the cell monolayers (0 h) and at 20 h post-scratch. Scale bars: 100 µm. (**C**,**D**) Percent open wound of the field view area occupied by (**C**) KRAS and (**D**) NRAS overexpressing NIH3T3 cells at 20 h post-scratch vs. time point 0 h. (**E**–**J**) Western blotting detection of epithelial-mesenchymal transition (EMT) markers (**F**,**I**) E-cadherin and (**G**,**J**) vimentin protein expression levels in NIH3T3 cells transfected with KRAS or NRAS expression constructs. Band intensities were normalized against GAPDH or total protein. Data presented are representative of three independent trials in triplicates and expressed as mean (standard deviation). ** *P* ≤ 0.01, *** *P* ≤ 0.001. EMT, epithelial-to-mesenchymal transition.

Scratch wound assays were performed to investigate the effect of the rare KRAS and NRAS variants on the migratory capacity of NIH3T3 cells. Cells overexpressing the three non-canonical/novel KRAS mutants each showed a significant increase in migration rates compared to wild-type KRAS and empty vector controls, akin to rates observed in cells expressing the constitutively active KRAS G12D variant (Figure 2A,C). Meanwhile, cells transfected with the novel variant NRAS A11V migrated into the wound gap at rates comparable to that of cells transfected with wild-type NRAS, a canonical NRAS G12D and vector-only control (Figure 2B,D). This implies that the NRAS A11V mutation does not affect the migratory capacity of NIH3T3 cells.

### 3.4. KRAS Mutants G12S, A59T, and Y137C Upregulate the Mesenchymal Marker Vimentin

At the molecular level, the increase in migration rates observed in cells overexpressing KRAS G12S, A59T, and Y137C may be due in part to their capacity to induce the process of epithelial-mesenchymal transition. To confirm this hypothesis, protein expression levels of the epithelial marker E-cadherin and the mesenchymal marker vimentin in transfected HCT116 cells were assessed by Western blot analysis. Band intensities normalized against GAPDH or total protein of the cell lysates visualized in pre-stained gels showed comparable E-cadherin levels among the empty vector control, wild-type KRAS, and non-canonical/novel KRAS mutant-transfected cells (Figure 2E,F). However, a marked increase in vimentin expression was observed in cells expressing KRAS G12D as well as KRAS G12S, A59T, and Y137C compared to wild-type KRAS and empty vector controls (Figure 2G). These results are consistent with results observed in scratch wound assays and suggest that G12S, A59T, and Y137C all promote cell migration, at least partly by inducing partial EMT.

The ability of the novel NRAS A11V mutation to induce EMT was similarly assessed in transfected HCT116 cells. No evident changes in E-cadherin expression was observed in cells transfected with NRAS A11V compared to wild-type NRAS-expressing cells (Figure 2H,I). Meanwhile, an apparent decrease in vimentin protein expression was observed in cells transfected with NRAS A11V versus wild-type NRAS expressing cells, similar to levels measured in cells expressing the canonical mutant NRAS G12D (Figure 2J). These results corroborate the lack of observable changes in cell motility in cells overexpressing NRAS A11V.

### 3.5. Cells Expressing KRAS and NRAS Mutants Display Altered F-Actin Cytoskeletal Organization

The onset of cellular transformation is known to be influenced by the deleterious impact of activating KRAS mutations on actin organization [34]. Non-transformed NIH3T3 fibroblasts typically display a flattened, spread-out phenotype indicating their static nature and adherence to the culture substrate [35]. In contrast, transformed NIH3T3 cells harbor phenotypic changes evidenced by smaller, rounder or more elongated, and more refractile cellular bodies that is characteristic of poor surface adhesion [36]. Cell depolarization is also apparent in transformed cells as indicated by lamellipodia formation and pronounced cellular protrusions [37].

**Figure 3:**
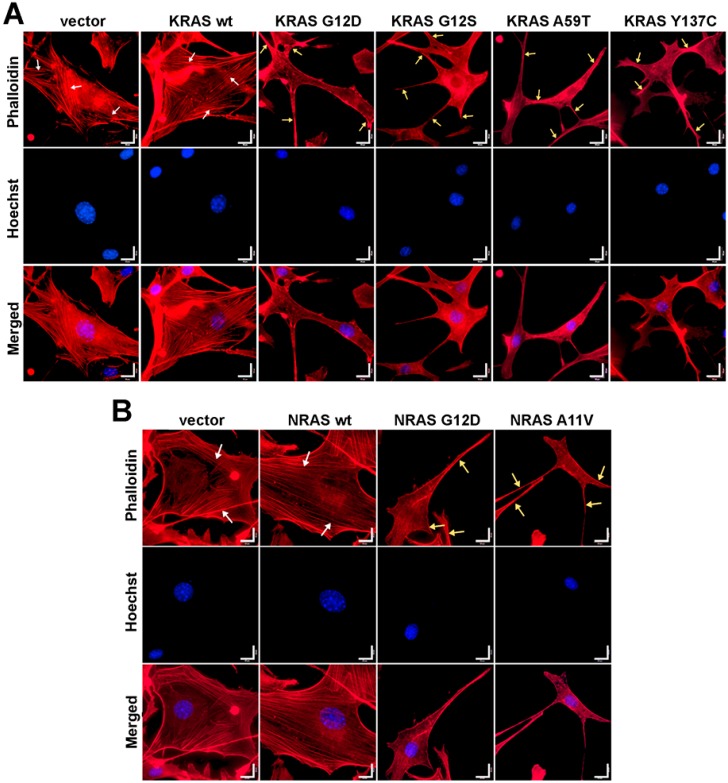
Expression of KRAS and NRAS mutants altered the cytoskeletal organization of NIH3T3 cells. Fluorescent images showing the F-actin cytoskeletal organization of NIH3T3 cells co-transfected with empty vector (i.e., pTargeT) and (**A**) KRAS or (**B**) NRAS expression constructs. Prominent and highly organized stress fibers (**white arrows**) are present in the empty vector setups as well as in cells transfected with wild-type KRAS and NRAS constructs. In contrast, cellular protrusions (lamellipodia, **yellow arrows**) were observed in NIH3T3 cells transfected with KRAS and NRAS canonical and novel mutant constructs. Cytoplasmic shrinkage and more diffused cytoskeletal actin were also observed in the mutant setups (phalloidin: F-actin; Hoechst: nuclei). Scale bars: 25 µm.

To determine if cytoskeletal changes similar to those caused by the canonical KRAS and NRAS G12D mutants can also be observed in cells expressing the rare KRAS and NRAS mutations, filamentous actin of fixed and permeabilized NIH3T3 cells was visualized through fluorescence-conjugated phalloidin staining. Cells transfected with empty vector control and wild-type KRAS or NRAS exhibited stable and prominent actin filaments with well-oriented parallel stress fibers (Figure 3). In contrast, cells overexpressing the KRAS G12S, A59T, and Y137C (Figure 3A) and NRAS A11V (Figure 3B) mutants all displayed gross morphological changes in cytoskeletal organization, as evidenced by cytoplasmic shrinkage and highly disorganized actin filaments. Multiple cellular protrusions including filopodia and lamellipodia were also observed around the cell periphery (Figure 3A,B). These cytoskeletal structures are indicative of highly motile cells with dynamic actin networks.

### 3.6. KRAS and NRAS Mutants Promote Cell Rounding, Refringency, and Cytoplasmic Shrinkage

**Figure 4:**
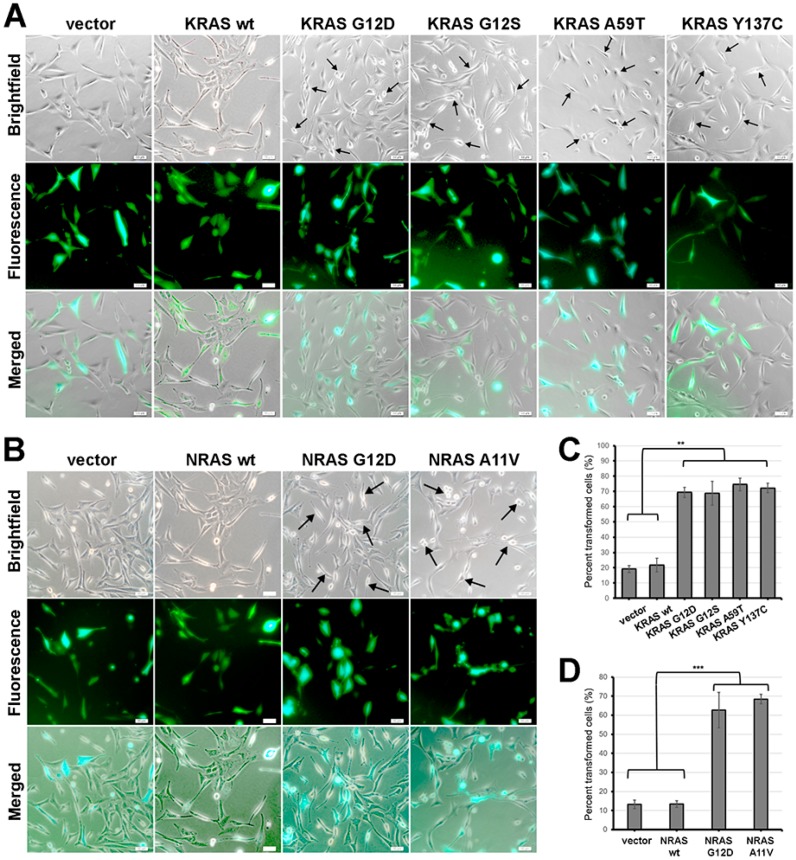
Expression of KRAS and NRAS mutants altered the gross cellular morphology of NIH3T3 cells. (**A**,**B**) Representative micrographs showing the cellular morphology of NIH3T3 cells co-transfected with empty vector (i.e., pTargeT) and (**A**) KRAS or (**B**) NRAS expression constructs. Cells were co-transfected with empty pmR-ZsGreen1 to estimate transfection efficiency. Arrows indicate cells with smaller, rounder, and more refringent morphology in contrast to empty vector and wild-type control cells which are flatter and more spread out. Scale bars: 25 µm. (**C**,**D**) Quantification of NIH3T3 cells overexpressing (**C**) KRAS and (**D**) NRAS mutants exhibiting apparent morphological alterations such as decreased size, refringency, and increased cellular protrusions. Data presented are representative of three independent trials and expressed as mean (standard deviation). ** *P* ≤ 0.01, *** *P* ≤ 0.001.

In addition to changes in actin organization, cellular morphology is another important phenotype which can be indicative of oncogenic transformation. In the context of NIH3T3 cells, constitutive activity of oncogenic RAS has been linked to morphological transformation [38]. To determine if KRAS G12S, KRAS A59T, KRAS Y137C and NRAS A11V can also induce changes in the overall morphology of NIH3T3 cells, positively transfected cells overexpressing the RAS variants and co-expressing the reporter gene ZsGreen1 were observed under a brightfield microscope. No discernible morphological changes were observed in cells transfected with wild-type KRAS and NRAS compared to empty vector controls (Figure 4A–D). As with the other cancer hallmarks described above, this is consistent with previous reports that overexpression alone of normal RAS variants is insufficient to confer a transformed cellular phenotype in NIH3T3 cells [33]. On the other hand, a considerable proportion of cells expressing either KRAS G12S, A59T or Y137C (Figure 4A), or the rare NRAS A11V variant (Figure 4B), showed morphological changes characteristic of transformed fibroblasts. Transfected cells expressing the fluorescent protein marker ZsGreen1 with apparent morphological alterations such as decreased size, refringency, and increased cellular protrusions were then quantified. The results confirmed that a significant fraction of fibroblasts co-transfected with the KRAS G12S, A59T, and Y137C mutants (Figure 4C; *P* < 0.01) and the rare NRAS A11V variant (Figure 4D; *P* < 0.001) exhibit these features characteristic of highly motile cells. Overall, these findings are consistent with the cytoskeletal reorganization observed in cells expressing the non-canonical/novel KRAS and NRAS variants, thus reinforcing evidence of the transformative effect of the rare mutations on cellular morphology.

### 3.7. Effects of KRAS and NRAS Mutations on ERK Phosphorylation and ELK-1 Transactivation

**Figure 5:**
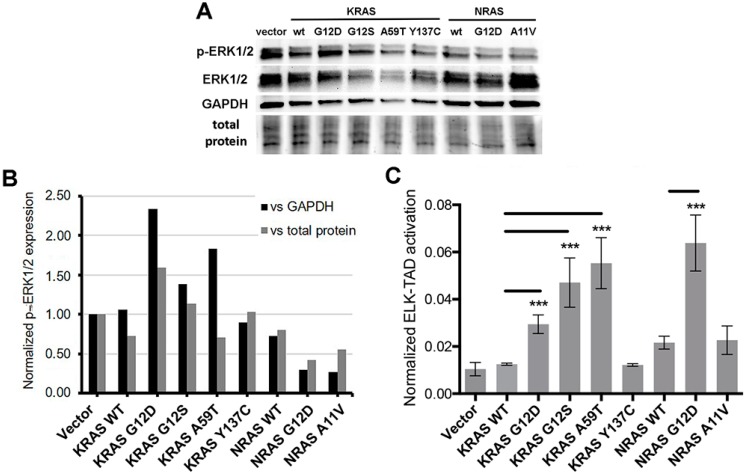
Effects of KRAS and NRAS mutants on ERK phosphorylation and ELK1 transactivation (**A**) Western blotting detection of pERK1/2 and ERK1/2 in NIH3T3 cells transfected with KRAS or NRAS expression constructs. (**B**) Quantification of pERK1/2 relative expression versus GAPDH or total protein. (**C**) Quantification of ELK-TAD activation. Data presented are representative of three independent trials and expressed as mean (standard deviation). *** *P* ≤ 0.001.

Oncogenic mutations frequently stabilize the RAS proteins in their GTP-bound form, resulting in constitutive activation of the mitogen-activated protein kinase (MAPK) pathway [39]. Hence, the ability of the novel KRAS and NRAS mutants to induce the activation of downstream targets of the RAS-RAF-MEK-ERK pathway was investigated through molecular assays. First, Western blot analysis was performed to examine the phosphorylation status of a well-established RAS downstream effector, ERK1/2 (Figure 5A). Band intensities normalized against GAPDH or total protein (visualized and quantified in pre-stained gel) per setup showed that the non-canonical KRAS G12S (*P* < 0.01) and A59T (*P* < 0.05) mutants expressed significantly higher levels of phosphorylated ERK1/2 similar to the canonical KRAS G12D variant compared to wild-type KRAS overexpressing cells (Figure 5B). On the other hand, p-ERK1/2 levels were comparable across NIH3T3 cells overexpressing the novel variants KRAS Y137C and NRAS A11V and their respective wild-type KRAS and NRAS controls (Figure 5B).

To confirm the activation of the MAPK pathway in response to overexpression of the KRAS mutants as evidenced by increased phosphorylation of ERK, an ELK-TAD luciferase reporter assay was performed. Phosphorylated ERK translocates to the nucleus and in turn activates one of its best characterized substrates, the ETS domain transcription factor ELK-1 by phosphorylating specific residues within its transcriptional activation domain (TAD) [40,41]. In this assay, a 293 cell line stably expressing a fusion protein linking ELK-TAD to Gal4 was used to monitor the effects of the KRAS and NRAS mutants on ELK1 transactivation. Phosphorylation of ELK-TAD in the fusion protein through the MAPK pathway drives firefly luciferase expression via Gal4. Normalized luciferase expression levels showed that NIH3T3 cells transfected with KRAS G12S and KRAS A59T significantly increased ELK-TAD activation compared to wild-type KRAS, similar to that observed in cells expressing the canonical KRAS G12D mutant (*P* < 0.001). In contrast, KRAS Y137C and NRAS A11V harbored comparable ELK-TAD activities to their respective wild-type KRAS and NRAS controls (Figure 5C). Overall, these results are consistent with the phosphorylated ERK1/2 readouts for the KRAS and NRAS mutants in the Western blot analyses, which imply that KRAS G12S and KRAS A59T confer an oncogenic phenotype through its downstream target ELK1. On the other hand, the novel KRAS Y137C and NRAS A11V mutants, which also mediated distinct oncogenic properties in the functional assays performed in this study, may be acting through other downstream targets to confer their respective aggressive phenotypic readouts to transiently transfected cells.

### 3.8. Bioinformatics-Based Modeling and Docking Simulations of the KRAS Mutants Predict Their Oncogenic Impact

The possible effect of the KRAS G12S, A59T, and Y137C mutations on the GTPase function of KRAS was analyzed using three bioinformatics prediction tools: PolyPhen-2, SIFT, and Align GVGD (Table 3). PolyPhen-2 uses physical and comparative considerations to predict the impact of an amino acid substitution based on the protein’s structure and function [26]. SIFT analyzes sequence homology and the physical properties of amino acids to predict whether an amino acid substitution affects protein function [27]. Align GVGD classifies missense substitutions in a scale ranging from enriched deleterious to enriched neutral by combining the biophysical characteristics of amino acids and protein multiple sequence alignments [28]. All three platforms predicted the canonical KRAS G12D and the non-canonical KRAS G12S and A59T mutations as possibly damaging mutations, with G12D classified as the most likely to interfere with function according to Align GVGD (Class C65; GD score: 93.77). On the other hand, the novel KRAS Y137C mutation yielded conflicting results from the three prediction platforms, with PolyPhen-2 reporting the mutation as benign, and Align GVGD classifying it as a Class C65 mutation similar to KRAS G12D, but with more damaging functional impact (GD score: 193.72) (Table 3). The impact of these four KRAS mutations was then investigated further by modeling and assessing the effect of each variant on overall protein structure. Docking simulations of each modeled structure with GTP was also done to determine the effects of each KRAS mutation on binding affinities with its natural ligand.

**Figure 6:**
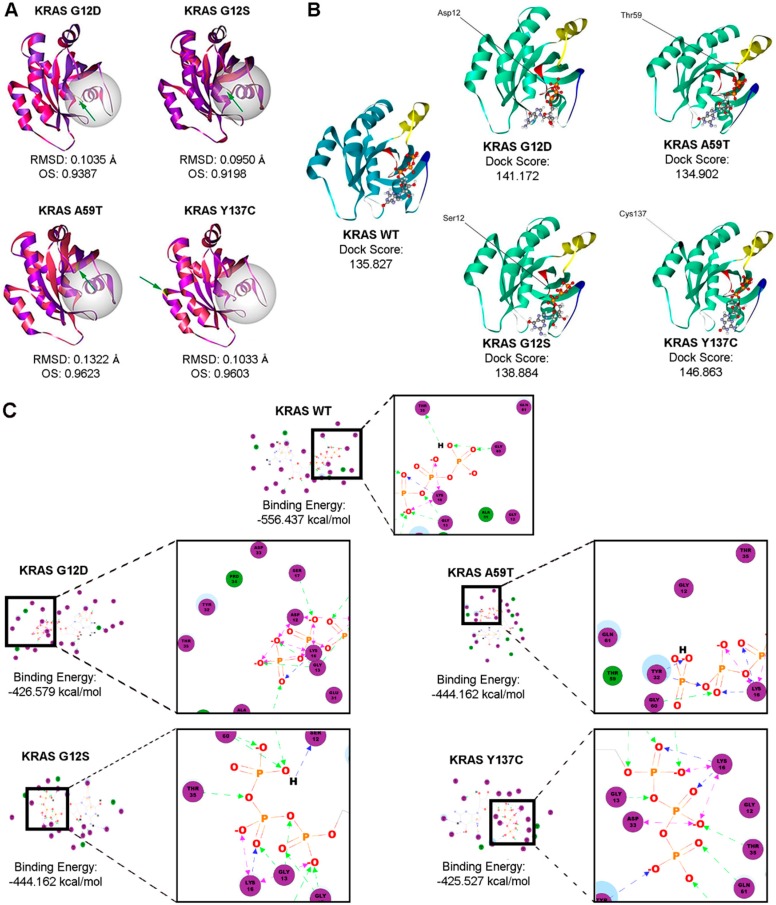
Bioinformatics-based modeling and docking simulations of the KRAS mutants predict their potential oncogenic impact. (**A**) KRAS mutants (pink) superimposed with the KRAS wild-type variant (purple) modeled by the Homology Modeler module of the Accelrys Discovery Studio 2.5.5 bioinformatics prediction platform. The position of each mutation is emphasized by the green arrow, while the GTP-binding pocket critical for the GTPase activity of KRAS is highlighted by the white sphere. (**B**) Docking simulations of wild-type and mutant KRAS with GTP using the LigandFit algorithm. Highlighted in the ribbon structures are the p-loop of KRAS (**red**), Switch I (**yellow**), and Switch II (**blue**), all of which comprise the active site of the KRAS protein. (**C**) The corresponding 2D-interaction diagrams from the resulting best poses are shown, with the highlighted amino acid interactions inset. One-way green, blue or black arrows between the residue and ligand indicate H-bonding, while two-way pink arrows represent charged interactions. RMSD, root mean square distance; OS, overall similarity; GTP, guanosine triphosphate.

The structural models of the KRAS non-canonical mutants G12S and A59T, and the novel mutant Y137C were generated using the Homology Modeler module of the Accelrys Discovery Studio 2.5.5 bioinformatics prediction platform. The structure of each resulting mutant model was superimposed to the wild-type KRAS structure, and both the chain root-mean-square distance (RMSD) and Overlay Similarity (OS) were computed (Figure 6A). RMSD values for mutant models overlaid to the wild-type structure did not exceed 0.15 as expected, since a single base change does not normally alter the protein structure significantly. The non-canonical KRAS A59T mutant had the highest RMSD, suggesting the highest deviation from the wild-type KRAS structure. However, the lowest OS was observed in the KRAS G12S model (OS: 0.9198). This implies that although the C-alpha carbons of the KRAS A59T mutant resulted in the most significant deviation from the C-alpha carbon backbone of the wild-type KRAS, the KRAS G12S variant had a greater impact at the atomic level, extending beyond the C-alpha carbon and possibly affecting even the orientation of the side chains, especially those at the GTP-binding pocket. Indeed, KRAS G12D had the next lowest OS of 0.9387, suggesting that amino acid substitutions at that particular position potentially alter the protein structure subtly, consequently affecting function.

Additionally, the effect of the KRAS mutations on ligand binding with GTP was assessed using the LigandFit molecular docking module. Figure 6 shows the resulting best poses based on the highest dock score (Figure 6B) and the two-dimensional interaction diagrams with the corresponding binding energies (Figure 6C, Supplementary Figure S2). As interaction of amino acid residues T35 and G60 of KRAS with the γ–phosphate of GTP stabilize the protein-ligand interaction in the active state, examining the changes in predicted interactions between these key switch residues and GTP in the context of each KRAS mutant may provide auxiliary insight on the impact of each mutation [42]. Compared to the wild-type KRAS two-dimensional interaction diagram, the KRAS G12D canonical mutation seemed to induce a charge-based interaction between acidic D12 and the gamma phosphate, leaving T35 with no direct interactors (Figure 6C). Such shifts in atomic interactions likely cause the functional impact of the G12D canonical mutation on KRAS intrinsic GTP-hydrolysis, consistent with previous reports as increased charged interactions between amino acid residue D12 and GTP theoretically prolongs the retention of KRAS in the GTP-bound active state [42]. The KRAS G12S mutation, on the other hand, though occurring in the same position, does not seem to alter the predicted binding interactions significantly, but was still able to mediate an increased H-bond-mediated interaction with GTP (Figure 6C). The KRAS A59T mutant also resulted in a marked shift in amino acid interactions, displacing T35 and inducing H-bonding with Y32 instead (Figure 6C). KRAS Y137C also seemed to have minimal effect on binding, but still caused some shift in binding, with T35 now forming H-bonds with the beta-phosphate instead of the gamma-phosphate (Figure 6C). Overall, these simulated structural alterations and disruption of important molecular interactions represent the possible functional role of mutations in the KRAS coding regions to which the observed aggressive phenotypic changes in the cancer hallmark assays performed in this study may be attributed.

### 3.9. Bioinformatics-Based Modeling of the Novel NRAS A11V Mutant Predicts Larger Changes in Overall Protein Structure but Higher Similarity to NRAS-WT than G12D

The effects of the novel NRAS A11V mutation compared to the canonical NRAS G12D mutation were also predicted using PolyPhen-2, SIFT, and Align GVGD. In all platforms, A11V was shown to have damaging effects on protein function similar to G12D (Table 3). Thus, further investigations on the impact of this novel NRAS A11V mutation were performed through modeling and assessment of its effects on overall protein structure.

**Figure 7:**
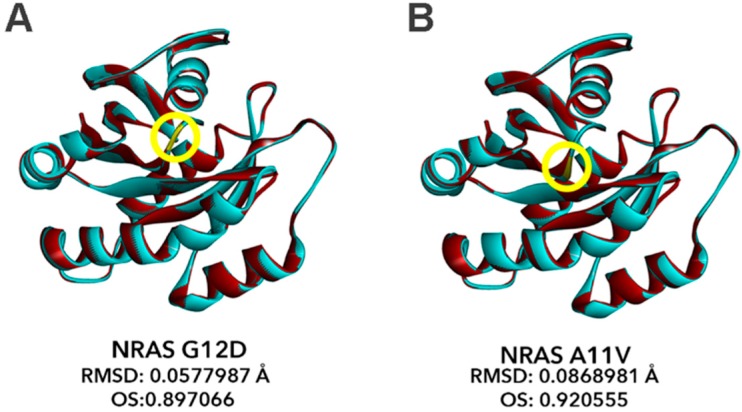
Bioinformatics-based modeling of the NRAS mutants predict their oncogenic impact. The canonical NRAS G12D mutant (**A**) and novel NRAS A11V mutant (**B**) (**red**) superimposed with the NRAS wild-type variant (**blue**) modeled by the Homology Modeler module of the Accelrys Discovery Studio 2.5.5 bioinformatics prediction platform. The position of each mutation within the NRAS GTP-binding pocket critical for GTPase activity of NRAS is indicated by the yellow encircled residues. RMSD and OS values of each NRAS mutant model versus NRAS WT as calculated using the BIOVIA Discovery Studio Visualizer is also indicated. RMSD, root mean square distance; OS, overall similarity.

The structural models of the canonical mutant NRAS G12D and the novel mutant NRAS A11V were generated using the Alignment Mode of the SWISS-MODEL automated protein structure homology-modeling server. The resulting structures of each mutant were superimposed onto the wild-type NRAS structure using the BIOVIA Discovery Studio 2019 bioinformatics prediction platform, and the chain RMSD and OS were computed (Figure 7). Similar to the non-canonical/novel KRAS mutants, RMSD values for NRAS G12D (RMSD: 0.0578; Figure 7A) and A11V (RMSD: 0.0869; Figure 7B) models overlaid to the wild-type NRAS structure also did not exceed 0.15, pointing to insignificant changes in the overall protein structure of both variants. However, the RMSD predictions calculated a larger deviation from the wild-type NRAS structure in the novel A11V mutant than the G12D mutant. This implies that the C-alpha carbons of the NRAS A11V mutant resulted in a more significant deviation from the C-alpha carbon backbone of the wild-type NRAS compared to the canonical NRAS G12D mutation. On the contrary, OS calculations show that the percent similarity between the wild-type NRAS protein structures and the A11V mutant (OS: 0.921) is greater than that with the G12D mutation (OS: 0.897). This implies that, similar to KRAS, amino acid substitutions in codon 12 subtly alters the protein structure and predictably affects function to a greater extent than A11V.

This result is expected since oncogenic missense mutations in RAS do not tend to affect overall protein structure drastically to significantly impact protein function. Canonical mutations such as G12D and Q61K only perturb specific parts of the RAS protein structure that lead to hyperactivation [43]. Mutations in codons 12 and 13 tend to convert the small glycine side chain into a bulkier R group, which can sterically inhibit the interaction between RAS and the RAS-GTPase activating protein (RAS-GAP). This leads to RAS being left in a constitutively active state due to prolongation of the GTP-bound state. Since the mutant A11V is within the same region as the G12D mutation, it is possible that the substitution from alanine to valine, an amino acid with a bulkier side chain, will elicit similar effects as G12D, albeit to a lesser extent.

## 4. Discussion

In this study, the oncogenic impact of four non-canonical/novel KRAS and NRAS mutations identified from malignant CRC tumor samples [15] were investigated. The two non-canonical KRAS mutations, G12S in exon 2 (NM_004985.3: c.34G>A; p.12G>S; COSMIC ID: COSM517) and A59T in exon 3 (NM_004985.3: c.175G>A; p.59A>T; COSMIC ID: COSM546) were previously reported in the COSMIC database, but have yet to be investigated for their functional effects upon extensive literature review. The KRAS G12S mutation has been reported to occur in 4.9%–5.7% of colorectal cancer samples harboring mutations in the KRAS gene [44]. The KRAS A59T mutation has been detected in 24 tissue samples from the large intestine [44], stomach [45], salivary gland [46], lung [44], and cervix [47], of which 13 cases (54%) were identified in CRC [44]. The novel mutants KRAS Y137C (NM_004985.3: c. 410A>G; p.137Y>C) and NRAS A11V (NM_002524.5: c. 32C>T; p.11A>V) have not been previously reported to occur in CRC and as such, their functional impact and phenotypic consequences also remain unknown.

Mutations in the RAS gene have been described in numerous studies, and mutant RAS has since been validated as a driver of tumor initiation and maintenance [8]. Mutations in KRAS hotspots impair the intrinsic activity of RAS by locking the mutant versions of the protein in a GTP-bound active conformation or prohibiting the binding of GTPase-activating proteins (GAPs) that facilitate GTP hydrolysis. This leads to the constitutive activation of downstream signaling cascades, including the MAPK pathway and PI3K/AKT pathway that is independent of ligand-induced activation from EGFR [48]. This, in turn, results in uncontrolled cellular proliferation, eventually leading to tumor formation. The mutational status of KRAS is also highly concordant between primary tumor formation and metastasis, suggesting a role in the early process of carcinogenesis [48]. Although less studied functionally and mechanistically, NRAS mutations have been associated with worse prognosis and overall survival when compared to KRAS mutant- or wild type KRAS-driven metastatic CRC [49]. Further, patients who are carriers of NRAS mutations (7.7%; 1/13) and are wild type for KRAS were shown to respond less to cetuximab treatment compared to NRAS wild type carriers (38.1%; 110/289). This demonstrates the utility and importance of including NRAS, as well as other downstream effectors of EGFR such as BRAF and PIK3CA, in mutational profiling especially among patients who are wild type for KRAS [5].

In silico analyses show that similar to the canonical KRAS G12D and NRAS G12D mutations, the non-canonical KRAS G12S and A59T mutations as well as the novel NRAS A11V mutation have high potential functional impact, based on three amino acid substitution analysis tools: PolyPhen-2, SIFT, and Align GVGD. Further, the novel KRAS Y137C mutation has a higher Grantham Difference (GD) score compared to KRAS G12D and was predicted to be in the highest class of mutations potentially altering protein function, according to Align GVGD. These results suggest that the rare mutations cause significant changes in RAS protein structure due to their critical positions within the protein. Protein structure modeling using the Homology Modeler protocol of Discovery Studio further reveals KRAS A59T as the mutant with the highest deviation from wild type by RMSD. The canonical mutant KRAS G12D, as expected, and the non-canonical mutant KRAS G12S, on the other hand, have the lowest overlay similarity to wild type, suggesting subtly nuanced effects on protein structure conferred by these mutations. Molecular docking simulations and analysis of ligand-receptor interactions between GTP and the KRAS mutants revealed marked differences in amino acid interactions. G12S did not mimic the charge-based shift in atomic interactions facilitated by G12D, most likely due to serine being uncharged. A59T and Y137C had marked and minimal effects on predicted binding interactions, respectively. In silico structural modeling of the novel NRAS variant A11V vis-à-vis the canonical mutant NRAS G12D revealed insignificant changes in the overall protein structure of both variants. OS calculations further revealed closer similarity in the structures of wild type NRAS and A11V compared with G12D, pointing to more significant effects on function by the subtle structural changes in the latter.

Functional and morphological characterization confirmed the transformative effects of the non-canonical and novel KRAS and NRAS mutations. Similar to NIH3T3 cells transfected with the canonical KRAS mutant G12D, cells transfected with KRAS G12S, A59T, and Y137C all displayed increased proliferation and migration rates. Increased vimentin expression in the epithelial cell line HCT116 was also observed upon transfection with the canonical KRAS G12D, the non-canonical KRAS G12S and the novel KRAS mutant Y137C, compared to KRAS wild type-expressing cells and vector only control. There was no apparent effect on E-cadherin expression which may be indicative of partial EMT. Cells expressing the canonical mutant NRAS G12D and the novel mutant NRAS G11V both showed no apparent effects on proliferation, migration and EMT. Instead, both NRAS mutants conferred increased resistance to apoptosis in HCT116, compared to wild type NRAS and vector only control. On the other hand, the canonical KRAS G12D, the non-canonical KRAS G12S and KRAS A59T, and the novel KRAS Y137C, did not cause resistance to apoptosis.

In contrast to the divergent effects of KRAS and NRAS mutants on the aforementioned hallmarks, all KRAS and NRAS mutants studied caused both altered F-actin organization and morphological changes when transfected into NIH3T3 cells, compared to wild type and vector only controls. Cytoplasmic shrinkage, disorganized actin filaments, and the presence of cellular protrusions were apparent in all mutant setups. Gross morphological changes such as cell rounding, refringency, and smaller cell size were also observed. Overall, these findings are consistent with the observed migratory phenotypes of the rare KRAS mutants and reveal an apparent effect of the rare NRAS A11V mutation on cell motility and adhesion which was not observed in the scratch wound assays. Cytoskeletal reorganization and pseudopodial formation are highly suggestive of a migratory phenotype, as these are among the first few steps taken by the cell to initiate movement [43]. The results therefore suggest that the expression of NRAS G12D and A11V may induce cells to initiate migration via their effects on the cytoskeleton but may not be involved in cellular motility, *per se.* Identification and elucidation of the signaling pathways involved in these phenotypic readouts can shed light on this apparent discrepancy. RAS-mediated cancer progression is known to engage multiple effectors. PI3Kα and PLC ε, for instance, can mediate cytoskeletal reorganization whereas RALGDS can regulate migration [50].

Differences in ability of the various mutants to signal via the downstream effector ERK and to activate ELK-1, a downstream nuclear target and well-characterized substrate of ERK, hint at distinct signaling pathways engaged by mutations in KRAS and NRAS. While cells transfected with KRAS G12S and KRAS A59T showed elevated p-ERK levels and significant ELK-1 activation similar to KRAS G12D, KRAS Y137C did not show any difference compared to KRAS wild type and vector only controls. NRAS wild type, NRAS G12D, and NRAS A11V did not have any effects on p-ERK levels but NRAS G12D appears to activate ELK-1 significantly.

Taken together, the results of this study point to overlapping and distinct phenotypic consequences of KRAS and NRAS mutations. Although preliminary, the p-ERK and ELK-1 activation assays provided initial data suggesting that different RAS mutants may engage different signaling pathways. Deviations from wild type structure, however minor, can affect amplitude and duration of binding to downstream effectors. While in silico modeling and ligand docking simulations were able to capture differences in predicted binding interactions, phenotypic characterization of the mutants only confirmed their oncogenicity and net effect on distinct cancer hallmarks. More mechanistic studies are warranted to identify the signaling pathways involved in mediating the different phenotypic readouts, to determine the differential oncogenic potencies of the mutants, and to determine if the structural constraints imposed by the different mutations may have altered association with downstream effectors. Mouse models, on the other hand, may help determine whether these mutations have a role in colorectal cancer initiation and progression, as well as resolve their functional consequences in vivo.

## Figures and Tables

**Table 1:** Primers used for generation and site-directed mutagenesis of Kirsten-RAS (KRAS) wild type and mutant constructs. (*) denotes where missense substitution was incorporated.

Designation	Sequence (5′-3′)
KRAS-WT-F	GCCGTCCTGAAGCTTCGCCGGATGACTGAATATAAACTTG
KRAS-WT-R	GTCGTGCCGCGGCCGCGTGCCGTTACATAATTACACACTTTG
KRAS-G12D-F	GCCGTCCTGAAGCTTCGCCGGATGACTGAATATAAACTTGTGGTAGTTGGAGCTGA*TGGCGTAGG
KRAS-G12S-F	GACTGTGGATCCATGACTGAATATAAACTTGTGGTAGTTGGAGCTA*GTG
KRAS-A59T-F	GGATATTCTCGACACAA*CAGGTCAAGAG GAGTAC
KRAS-A59T-R	GTACTCCTCTTGACCTGTTGTGTCGAGAATATCC
KRAS-Y137C-F	GGACTTAGCAAGAAGTTG*TGGAATTCCTTTTATTG
KRAS-Y137C-R	CAATAAAAGGAATTCCACAACTTCTTGCTAAGTCC

**Table 2:** Primers used for generation and site-directed mutagenesis of neuroblastoma-RAS (NRAS) wild type and mutant constructs. (*) denotes where missense substitution was incorporated.

Designation	Sequence (5′-3′)
NRAS-WT-F	ATGACTGAGTACAAACTGGTGGTGGTTGGAG
NRAS-WT-R	TTACATCACCACACATGGCAATCCCATAC
NRAS-G12D-F	ATGACTGAGTACAAACTGGTGGTGGTTGGAGCAGA*TGGT
NRAS-A11V-F	ATGACTGAGTACAAACTGGTGGTGGTTGGAGT*AGGTGG

**Table 3:** Predicted functional effects of KRAS G12S, A59T, and Y137C, and NRAS A11V.

Protein	Mutation	Classification	Polyphen-2 (score)	SIFT (score)	Align GVGD (GD Score)
KRAS	G12D	canonical	Possibly damaging (0.517)	Damaging (0.00)	Class C65 (93.77)
KRAS	G12S	COSMIC	Possibly damaging (0.682)	Damaging (0.01)	Class C55 (55.27)
KRAS	A59T	COSMIC	Possibly damaging (0.936)	Damaging (0.03)	Class C55 (58.02)
KRAS	Y137C	novel	Benign (0.353)	Damaging (0.00)	Class C65 (193.72)
NRAS	G12D	canonical	Possibly damaging (0.488)	Damaging (0.00)	Class C65 (93.77)
NRAS	A11V	novel	Possibly damaging (0.922)	Damaging (0.02)	Class C55 (64.43)

## Supplementary Materials

Supplementary Materials can be found at https://www.mdpi.com/2073-4409/8/12/1557/s1. Figures S1 and S2.
