# Supplementary material for: Non-Redundant and Overlapping Oncogenic Readouts of Non-Canonical and Novel Colorectal Cancer KRAS and NRAS Mutants
Source: Cells. 2019 Dec 3;8(12):1557. doi: 10.3390/cells8121557 (PMC6952822; doi:10.3390/cells8121557)
Supplement: Supplementary file 1 [file cells-08-01557-s001.pdf]

Non-Redundant and Overlapping Oncogenic Readouts of Non-Canonical and Novel Colorectal Cancer KRAS and NRAS Mutants

Krizelle Mae M. Alcantara [1,†], Joshua Reginald P. Malapit [1,‡,§], Ryan Timothy D. Yu [1,§], Jose Antonio Ma. G. Garrido [1], John Paul T. Rigor [1], Arlou Kristina J. Angeles [1,‖], Eva Maria Cutiongco-de la Paz [2,3] and Reynaldo L. Garcia [1,3,*]

[1] Disease Molecular Biology and Epigenetics Laboratory, National Institute of Molecular Biology and Biotechnology, University of the Philippines Diliman, Quezon City 1101, Philippines

[2] Institute of Human Genetics, National Institutes of Health, University of the Philippines Manila, Manila 1000, Philippines

[3] Philippine Genome Center, University of the Philippines System, Quezon City 1101, Philippines

* Correspondence: reygarcia@mbb.upd.edu.ph; Tel.: +63 2 9818500 Loc. 3953

† Present Address: Center for Gene Therapy, Abigail Wexner Research Institute, Nationwide Children's Hospital, 700 Children's Drive, Columbus, OH 43205, USA

‡ Present Address: Division of B Cell Immunology, German Cancer Research Center, Im Neuenheimer Feld 280, Heidelberg 69120, Germany

§ These authors contributed equally to this work.

‖ Present Address: Division of Cancer Genome Research, German Cancer Research Center and National Center for Tumor Diseases. Im Neuenheimer Feld 460, Heidelberg 69120, Germany

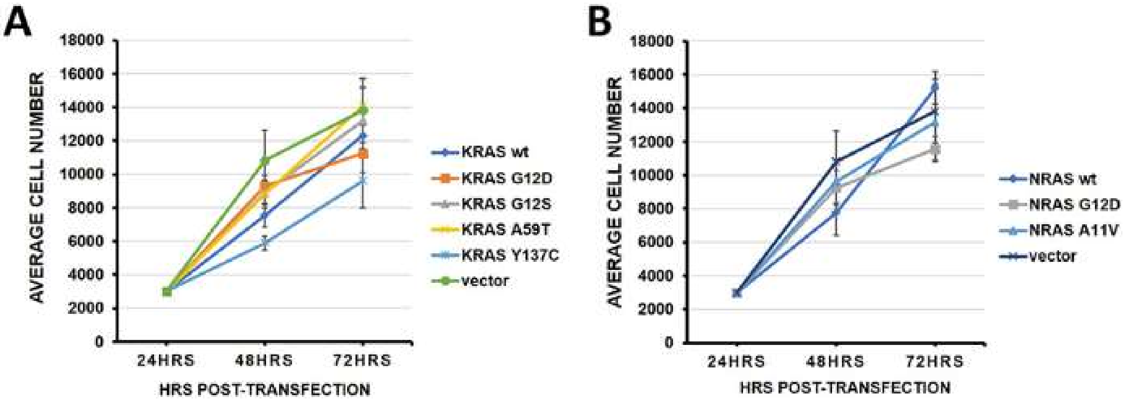

Supplementary Figure S1. No significant changes in cell proliferation rates were observed in NIH3T3 cells expressing KRAS and NRAS mutants when maintained in full-serum (10%) conditions. (A) Proliferation rate of NIH3T3 cells transfected with empty vector, wild type KRAS or KRAS mutant constructs. (B) Proliferation rate of NIH3T3 cells transfected with empty vector, wild type NRAS or NRAS mutant constructs.

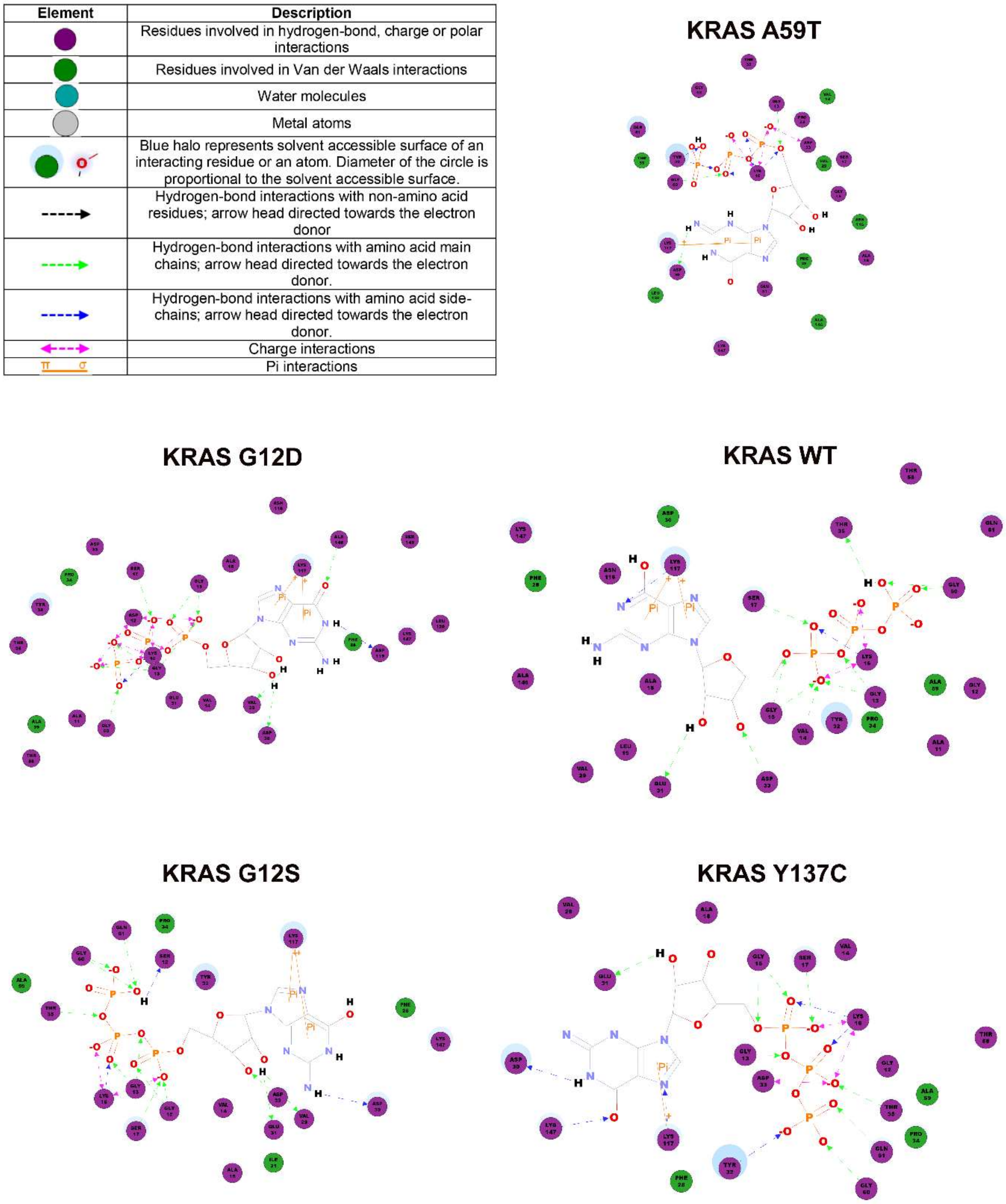

Supplementary Figure S2. The corresponding full, high-resolution 2D-interaction diagrams from the resulting best poses of the KRAS variants from Fig. 6C are shown with the protein-interaction legend.
